# The natural compound n-butylidenephthalide suppresses type II ovarian cancer cell growth by inducing ferroptosis

**DOI:** 10.7150/ijms.114086

**Published:** 2025-08-22

**Authors:** Shinn-Zong Lin, Chih-Yang Huang, Yueh-Min Lin, Rathinasamy Baskaran, Yu-Jung Lin, Wei-Wen Kuo, Dah-Ching Ding

**Affiliations:** 1 Bioinnovation Center, Buddhist Tzu Chi Medical Foundation, Hualien, Taiwan.; 2 Department of Neurosurgery, Hualien Tzu Chi Hospital, Buddhist Tzu Chi Medical Foundation, Hualien, Taiwan.; 3 Cardiovascular and Mitochondrial Related Disease Research Center, Hualien Tzu Chi Hospital, Buddhist Tzu Chi Medical Foundation, Hualien, Taiwan.; 4 Graduate Institute of Biomedical Sciences, China Medical University, Taichung, Taiwan.; 5 Department of Medical Research, China Medical University Hospital, China Medical University, Taichung, Taiwan.; 6 Center of General Education, Buddhist Tzu Chi Medical Foundation, Tzu Chi University, Hualien, Taiwan.; 7 Department of Medical Laboratory Science and Biotechnology, Asia University, Taichung, Taiwan.; 8 Department of Pathology, Changhua Christian Hospital, Changhua, Taiwan.; 9 School of Medicine, Chung Shan Medical University, Taichung, Taiwan.; 10 Department of Research, Taipei Tzu Chi Hospital, Buddhist Tzu Chi Medical Foundation, New Taipei, Taiwan.; 11 Department of Biological Science and Technology, College of Life Sciences, China Medical University, Taichung, Taiwan.; 12 Ph.D. Program for Biotechnology Industry, China Medical University, Taichung, Taiwan.; 13 School of Pharmacy, China Medical University, Taichung, Taiwan.; 14 Department of Obstetrics and Gynecology, Hualien Tzu Chi Hospital, Buddhist Tzu Chi Medical Foundation, Tzu Chi University, Hualien, Taiwan.; 15 Institute of Medical Sciences, Tzu Chi University, Hualien, Taiwan.

**Keywords:** ferroptosis, ovarian cancer, high-grade serous carcinoma, n-butylidenephthalide, cancer stem cells

## Abstract

Type II high-grade serous ovarian cancer (HGSOC) accounts for 80% of all ovarian cancers. Tumor metastasis and chemotherapy resistance are predominantly caused by cancer stem cells (CSCs). n-butylidenephthalide (BP) has showed promise as an anti-tumor drug in a variety of malignancies. The purpose of this study was to examine the ferroptosis influence of BP on HGSOC. CSCs were isolated from the HGSOC cell lines KURAMOCHI and OVSAHO by employing the CSC marker ALDH. The study assessed cell survival, proliferation, IC_50_ (the concentration at which 50% growth suppression occurs), terminal deoxynucleotidyl transferase (TdT) dUTP nick end labeling (TUNEL) assay, and Western blot analysis of ovarian cancer cells and CSCs. qPCR was performed to assess ferroptosis-related gene expression. Furthermore, animal studies were carried out using a mouse model with subcutaneous xenografted stemness-enriched ovarian CSCs and evaluated with an IVIS scan. In this *in vivo* investigation, control, BP with or without taxol or ferrostatin were intended as cancer treatments. The findings indicated that BP inhibits HGSOC growth via ferroptosis mediated by the GPX4 conventional and HMBOX-1 noncanonical pathways. Furthermore, the anti-tumor effects of BP and Taxol were significantly improved when tumor-bearing mice were treated with both simultaneously, compared to their sole therapy. BP may increase the susceptibility of ovarian cancer cells to Taxol, implying its potential to improve the therapeutic effects of this standard ovarian cancer treatment.

## Introduction

Ovarian cancer is one of the most fatal gynecological cancers among women. In Taiwan, statistics from 2002 to 2015 revealed that ovarian serous carcinoma was the most frequent histology, accounting for 30.9% of ovarian cancer patients 1, 2. Recent advances in pharmacological therapy have resulted in a moderate improvement in ovarian cancer survival rates. Nonetheless, even with screening programs in nations like the United Kingdom and the United States, the fatality rates associated with ovarian cancer remain the same 3, 4.

Based on histological, molecular, and genetic research, ovarian tumors have lately been divided into two major types to better understand their carcinogenesis. Mucinous carcinomas, endometrioid, clear cell, transitional cell, and low-grade serous carcinomas are all examples of type I malignancies. Type II consists of undifferentiated carcinomas, carcinosarcomas, and high grade serous ovarian carcinomas (HGSOC) 5, 6. Tumors are made up of cells with variable degrees of malignancy. Tumor growth is controlled by specialized, pluripotent, self-renewing cells with tumorigenic properties known as cancer stem cells (CSCs). These CSCs frequently resist traditional treatments, prompting the development of medications that specifically target them. CSCs can be identified and isolated for investigation in the most frequent type of ovarian cancer, HGSOC, utilizing aldehyde dehydrogenase activity (ALDH) as a marker 7-9.

The current strategy to ovarian cancer treatment consists of debulking surgery and adjuvant chemotherapy with platinum and paclitaxel. Despite this treatment, ovarian cancer has a significant recurrence rate. Resistance to existing chemotherapeutic drugs may develop, especially after many rounds of chemotherapy. Furthermore, the toxicity associated with these medications frequently prevents some patients from finishing their treatment 10, 11. This highlights the need for novel medications or techniques to improve the efficacy of existing ones. Taxol was an FDA approved cancer drug used to treat ovarian cancer. Ovarian cancer exhibits resistance to the taxol treatment through GPX4-mediated ferroptosis. GPX4 knockout in ovarian cancer cell OVCAR-3 reduced the cell viability and colony formation 12. Thus, there is a need for identifying a compound which could sensitize or act synergistically with taxol to induce cell death in the ovarian cancer cells.

Ferroptosis differs from apoptosis, necrosis, and autophagy in that it is dependent on intracellular iron. Extensive investigations have revealed the crucial function of ferroptosis in tumor suppression, so giving a new technique for cancer treatment, particularly in ovarian 13, 14. Ferroptosis is defined by the accumulation of lipid peroxides in cells, which causes oxidative damage and death. It differs from other types of cell death, such as apoptosis and necrosis. Ferroptosis occurs when cellular iron homeostasis is disrupted and reactive oxygen species (ROS) accumulate, notably lipid peroxides. This process is driven by the inhibition of important enzymes such as glutathione peroxidase 4 (GPX4) and ferroptosis suppressor protein 1 (FSP1), which play crucial roles in protecting cells against oxidative damage 15, 16. Jiang et al. have suggested that ferroptotic cancer cells have the potential to induce robust immune responses and improve antitumor immunity by activating immunogenic cell death 17. This means that ferroptosis is a key mechanism in ovarian cancer treatment. There is hope for a new approach to treating ovarian cancer if the associated mechanism is better understood and the target of action is defined.

*Angelica sinensis*, a popular Chinese herbal medication, has been used to treat coughs, headaches, angina, and muscle strengthening. N-butylidenephthalide (BP) is an active component of *A. sinensis*' chlorinated layer 18. BP has been investigated for its therapeutic potential in a variety of cancers, including brain tumors 19, gastric cancer 20, and liver cancer 21, primarily by apoptotic cell death. The effect of BP on HGSOC death through ferroptosis has yet to be determined. Ferroptosis-based cancer therapy is emerging as a unique approach to cancer treatment. This study seeks to determine whether BP can eradicate HGSOC CSCs through ferroptosis. In this study, we describe how BP induces ferroptosis in ovarian cancer and CSCs.

## Materials and Methods

### Chemicals and antibodies

BP (ThermoFisher Scientific) was dissolved in a vitamin K solution (Sigma-Aldrich). The stock solution had a concentration of 200 μg/μl. Antibodies used included GPX1 (#3286), GPX4 (#52455) (Cell Signaling Technology, Danvers, MA, USA), GPX2 (sc-133160), GPX3 (sc-58361), and GAPDH (sc-32233) (Santa Cruz Biotechnology, Santa Cruz, California, USA).

### Cell culture

The HGSOC cell lines (KURAMOCHI and OVSAHO) used in this investigation were obtained from Japan Cell Bank. Both cell lines had gene expressions that mimicked HGSOC 22. The cell line was cultured in Dulbecco's Modified Eagle Medium (DMEM) (Sigma, St. Louis, MO, USA) with 10% Fetal bovine serum (FBS), 0.1% non-essential amino acids (NEAA), 2 mM L-glutamine, and 1% penicillin-streptomycin. The cells were incubated at 37 °C with 5% CO_2_.

### Isolation of ovarian CSC by flow cytometry

We isolated ALDH+ cells from the KURAMOCHI and OVSAHO cell lines using fluorescence-activated cell sorting (FACS) 23. The Aldefluor test kit (Stem Cell Technologies, Cambridge, MA, USA) was used to measure ALDH activity. The activated ALDEFLUOR reagent was trypsinized and treated with cells for 50 minutes at 37°C. Cells were treated with the inhibitor DEAB as control cells to detect ALDH+ and ALDH- cell populations. The stained cells were then examined and analyzed using a BD FACSVerse flow cytometer (BD Biosciences, San Jose, California, USA). The BD FACSAria Fusion flow cytometer (BD Biosciences) was used to sort ALDH+ cells. After sorting, ALDH+ and ALDH- cells were cultured in the aforesaid culture media for no more than five passes. According to our analysis, the percentage of ALDH+ cells was greater than 80% within five passages.

### Assessment of cell viability

Cell viability was evaluated using the XTT assay (Biological Industries Ltd., Kibbutz Beit HaEmek, Israel) according to the manufacturer's instructions. We seeded 2 × 10^3^ cells/cm^2^ in 96-well plates with different doses of BP (0, 25, 50, 75, 100 and 125 μg/ml) for KURAMOCHI and OVASAHO cells for 48 hours. The half-maximal inhibitory concentration (IC_50_) for both cell types was then calculated. The previous literature outlined the four-parameter logistic regression (4PL) method that was applied 24. The equation is written as follows: Y=d+(a-d)/(1+(X/c)b), where Y represents the response and X represents concentration. The variables a and d represent the curve's bottom and top, respectively. The variable b is the slope factor, and c is the concentration that corresponds to the response halfway between a and d 25. The XTT solutions and N-methyl dibenzopyranzine methyl sulfate (PMS) were promptly defrosted in a water bath set to 37 °C. To each 100-μL culture in 96-well plates, 50 μL XTT/PMS was applied. After 2-5 hours of incubation at 37 °C, plates were spectrophotometrically examined to determine the optical density of the solutions at 450 nm (650 nm being the reference wavelength). All experiments were performed three times in triplicates.

### siRNA transfection

Ovarian cancer cells were transfected with siHMBOX1 sequence: GGAAGTTCATATGGGAATA (siHMBOX1-1: 25 nM and siHMBOX1-2: 50 nM) (synthesized by Invitrogen) and siNC (scrambled) using Lipofectamine^TM^ 2000 according to the manufacturer's instructions.

### Quantitative real-time PCR

2 × 10^5^ KURAMOCHI cells were seeded per well in a 6-well plate. KURAMOCHI cells were treated with BP (25 and 50 μg/ml) for 48 hrs and harvested. Gene expressions in the treatment group were measured using Reverse Transcription Quantitative Polymerase Chain Reaction (RT-qPCR). Glyceraldehyde 3-phosphate dehydrogenase (GAPDH) served as an internal control. Table 1 shows the primer sequences used. In brief, real-time PCRs were carried out using FastStart Universal SYBR Green Master (ROX, Roche, Indianapolis, IN, USA) and a qPCR detection system (ABI Step One Plus system, Applied Biosystems, Foster City, CA, USA). Expression levels of each target gene were determined using the 2-ΔΔCt method 20. Each gene of interest was measured three times per experimental sample. Furthermore, the experiments were performed three times.

### Western blot analysis

At the end of treatment cells were washed with ice cold Phosphate-buffered saline (PBS) and lysed with Radioimmunoprecipitation (RIPA) lysis buffer system (sc-24948, Santa Cruz). Protein content was estimated and equal volume of protein (50 μg) was separated using 10-12 % sodium dodecyl sulfate-polyacrylamide gradient gel at 120 V for 90 mins. Following electrophoretic separation, the proteins were transferred to a polyvinylidene difluoride membrane (Bio-Rad) at 100 V for 60 mins at 4 °C. The membrane was then blocked with 5% nonfat dry milk in TBST at room temperature, then washed with Tris-buffered saline with 0.1% Tween 20 (TBST) for 5 mins thrice. Blots were incubated with respective primary antibody (1:1000) at 4°C overnight in rocker. The blots were then washed with TBST for 5 mins thrice and incubated with respective secondary antibody (1:5000) conjugated with horseradish peroxidase (HRP) at room temperature. Blots were washed with TBST for 10 mins thrice HRP signals were identified using an electrochemiluminescence kit (Promega, Fitchburg, WI, USA). GAPDH proteins (Santa Cruz Biotechnology, Santa Cruz, California, USA) served as internal controls 26.

### ROS and Lipid peroxidation measurement

ROS measurement was performed using DCFDA/H_2_DCFDA - Cellular ROS Assay Kit (ab113851, Abcam) according to the manufacturer's instructions. In brief, each 2 × 10^4^ cells (KURAMOCHI ALDH^+^ and OVSAHO ALDH^+^) were seeded with per well in 96-well plate. Following the drug treatment (BP 50 μg/ml, Sorafenib-20μM, Ferrostatin-20μM) for 1 hr, media was removed, washed with PBS, 100 μl of 25 μM DCFDA was added to the cells and incubated for 45 mins at 37°C. Fluorescence intensity in different treatment groups was read using BioTek Synergy fluorescence microplate reader (excitation=485 nm; emission=535 nm). All experiments were performed three times in triplicates.

Lipid peroxidation in the ovarian cancer cells (KURAMOCHI and KURAMOCHI ALDH^+^) was measured as previously described method 27. Briefly, 2 × 10^5^ cells were seeded per well in a 6-well plate. The cells were treated with treatment BP (50 μg/ml) and Ferrostatin (20μM) for 48 hr and collected for the analysis. Then cells were resuspended in 500 μl PBS containing DHR123 and C11-BODIPY (581/591) and incubated for 10 minutes at 37 °C. Cells were then resuspended in 500 μl of PBS containing SytoxBlue and analyzed by flow cytometry (BD Biosciences).

### Annexin V/PI staining assay

Cells were grown in the presence or absence of BP at IC_50_. Apoptotic cells were analyzed using annexin V-FITC detection kits (BD Pharmingen™) as per manufacturer's instructions. Cells were collected, treated with 5 µl of FITC Annexin V and 5 µl of PI, and incubated for 15 minutes in the dark. After incubation, analyze the cell mortality by flow cytometry within an hour.

### Assessment of BP activity *in vivo*

The animal experiment techniques were approved by the Buddhist Tzu Chi General Hospital's Animal Research and Care Committee (109-62). All procedures followed the National Institutes of Health's Guide for the Care and Use of Laboratory Animals. Non-obese, diabetic-severe combined immunodeficiency mice (NOD-SCID) (strain NOD.CB17-Prkdcscid/JTcu) acquired from Tzu Chi University were utilized in this study.

KURAMOCH ALDH+ cells had previously been transfected with luciferase. We employed Firefly Luciferase Lentivirus (Hygromycin) (Catalog #: 79692-H, BPS Bioscience, San Diego, CA) to transfect luciferase into KURAMOCHI ALDH+ cells. Approximately 20,000 cells per well were infected with 200,000 TU (Transduction Units) with firefly luciferase lentivirus using spinoculation. After 66 hours of transduction, the culture medium was replaced with a standard growth medium. The luciferase test was carried out using the ONE-Step^TM^ Luciferase assay system (BPS Bioscience, #60690), following the protocol given in the user handbook. The resultant cells are named KURAMOCH ALDH+/luc.

KURAMOCHI ALDH+/luc cells (1× 10^6^) were injected subcutaneously into the backs of female mice aged 4-5 weeks. After the tumors had grown to a volume of 50 mm^3^, mice were randomly divided into six groups (control, taxol (10 mg/kg), BP (200 mg/kg), BP (200 mg/kg) + Ferrostatin-1 (1 mg/kg in 1% DMSO), BP + taxol (5 mg/kg), BP + taxol (10 mg/kg); (n = 3 per group). BP was supplied via subcutaneous injection near the tumor location. Taxol and ferrostatin-1 were injected intraperitoneally. The control group was treated solely with the vehicle (DMSO). In contrast, the experimental group received a 5-day prescription of BP (200 mg/kg) in combination with other medicines. Taxol was supplied once weekly, completing three injections, while ferrostatin-1 was administered over a period of five days. After the IVIS CT imaging or the tumor reached a volume of 500 mm^3^, the mice were killed in a box filled with 100% CO_2_. Tumor samples were fixed in 4% paraformaldehyde prior to histological analysis.

### TUNEL assay

A small portion of tumor tissue stored in formalin solution was embedded in paraffin wax and cut into 6 μm thickness using microtome and fixed in a clean glass slide. The slide was deparaffinize and rehydrated. TUNEL assay was performed in the tissue section according to the manufacturer's instructions using TUNEL Assay Kit (Roche, IN, USA).

### Statistical analysis

Data are presented as the mean ± SD of at least three independent experiments. The Mann-Whitney U test was used to compare two independent variables, and one-way ANOVA with post-hoc analysis with the Bonferroni test was used to compare three independent variables. Statistical analysis was performed using GraphPad Prism 8 (La Jolla, CA, USA). P < 0.05 was considered a significant difference.

## Results

### Survival of cancer cells after BP treatment

**Figure 1:**
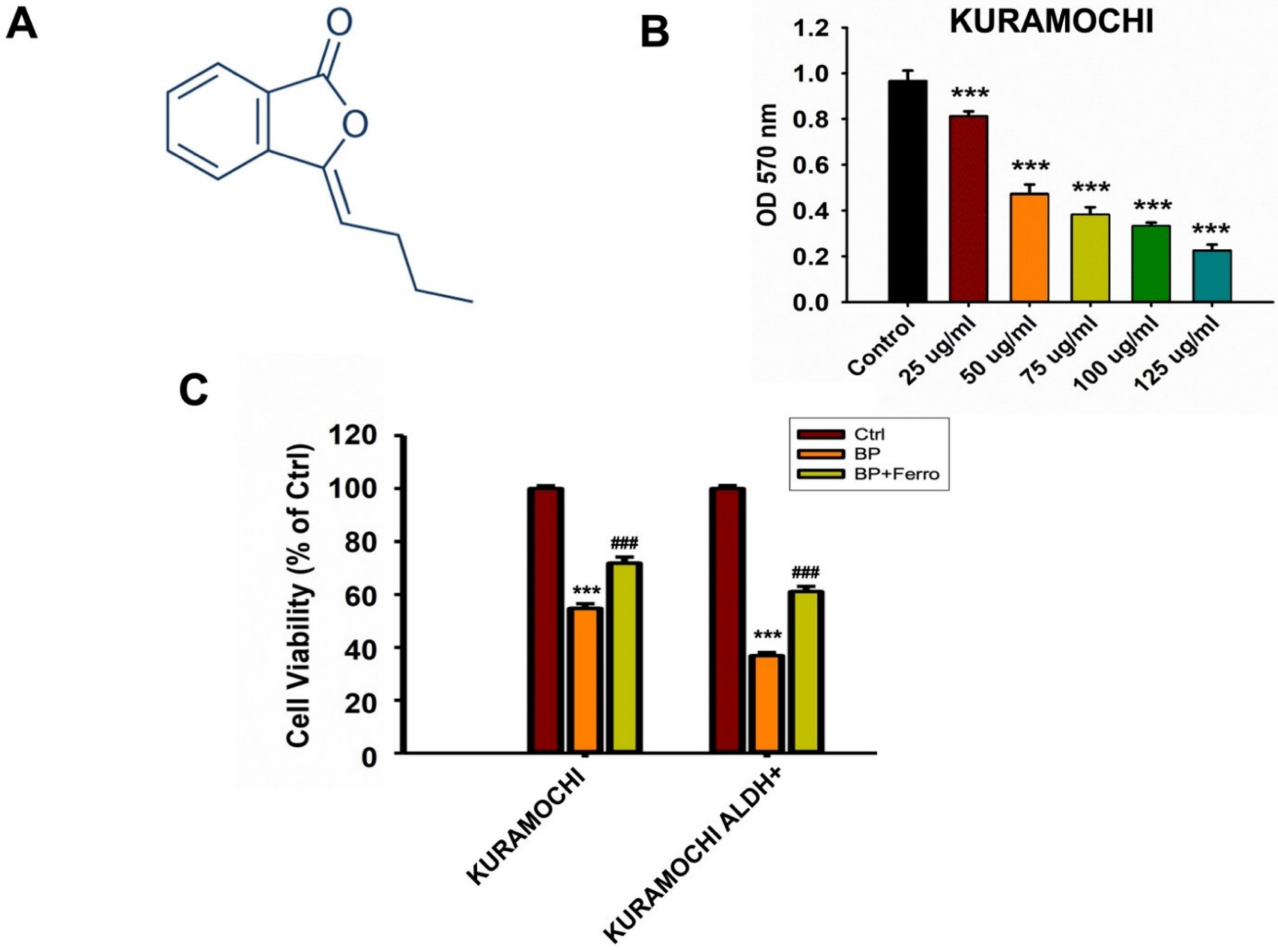
N-butylidenephthalide (BP) treatment reduced KURAMOCHI and OVSAHO cell survival. (A) Chemical structure of N-butylidenephthalide (B) An ELISA reader measured cell survival after KURAMOCHI cells were treated with different concentrations of BP (25, 50, 75, 100 and 125 μg/ml) at a wavelength of optical density (OD) 570 nm. ***p<0.001 vs control. (C) Cell viability of KURAMOCHI and KURAMOCHI ALDH^+^ cells, after treating BP (50 μg/ml) with or without ferrostatin (20 μM). ***p<0.001 vs control, ###p<0.001 vs BP.

Figure 1 shows how BP therapy affected the survival of KURAMOCHI and OVSAHO, as well as their stem-like cells (ALDH+). We found that N-butylidenephthalide (BP) treatment greatly decreased the survival and viability of KURAMOCHI and OVSAHO cells (Figure 1A). Co-treatments with ferroptosis inhibitors such as ferrostatin, sulfasalazine, and vitamin E were also investigated, and the results showed that they reversed cell survival (Figure 1B). ALDH+ ovarian cancer stem-like cells appeared to be more susceptible to BP than cancer cells, and treatment with ferrostatin restored their mortality (Figure 1C). These findings showed a potential therapeutic influence of BP on these ovarian cancer cells, specifically ferroptosis, a type of controlled cell death.

### BP suppresses GPX4 in KURAMOCHI cells

**Figure 2:**
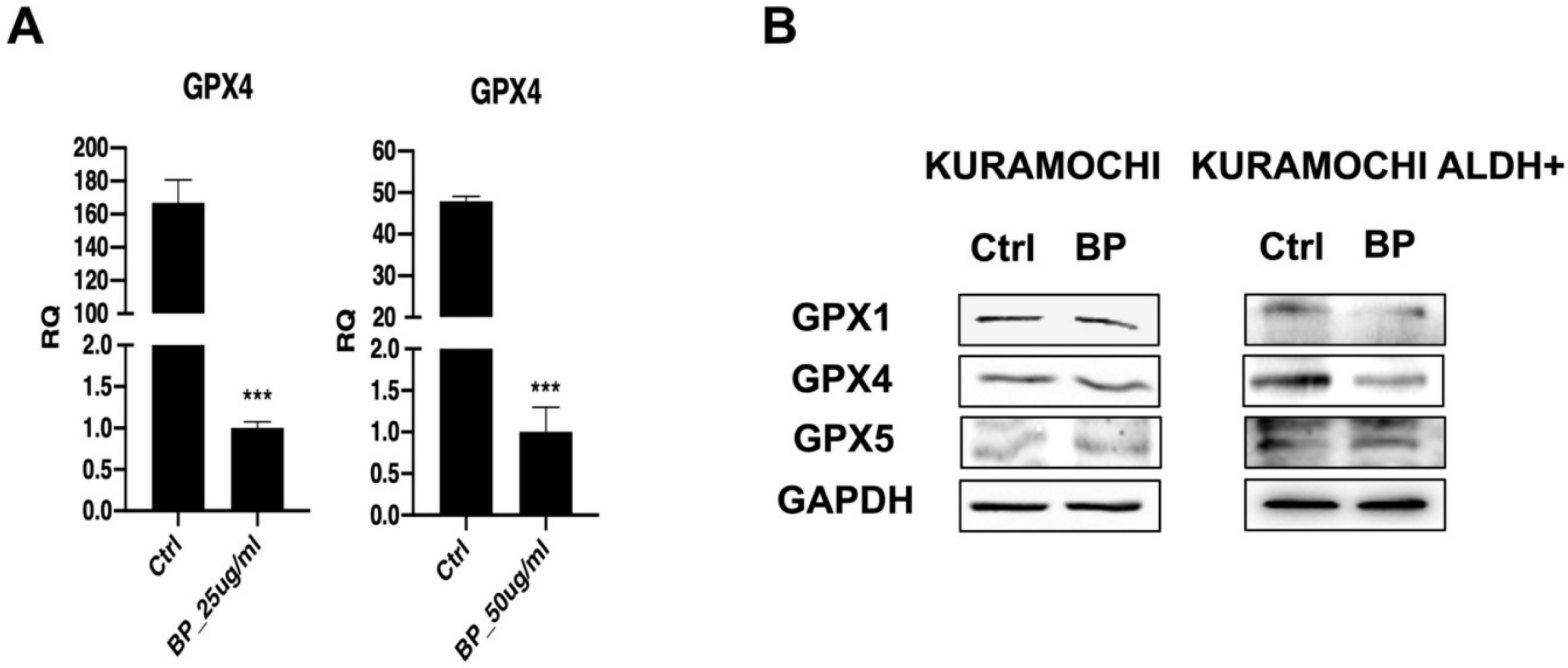
The expression of ferroptosis-related gene and protein in KURAMOCHI cells after n-butylidenephthalide (BP) treatment. (A) qPCR revealed *GPX4* expression in KURAMOCHI cells after treating BP 25 or 50 μg/ml for 48 hrs was decreased. ***p<0.001 vs control. (B) Western blot revealed reduced expression of both GPX1 and GPX4 proteins in KURAMOCHI ALDH^+^.

The effect of BP on the expression of the ferroptosis regulating gene GPX4 was explored further. The data imply that BP treatment reduced GPX4 gene expression at both the mRNA and protein levels in KURAMOCHI cells (Figure 2A and 2B). In addition, the study found that BP treatment reduced the expression of GPX1 and GPX4 proteins in KURAMOCHI ALDH+ CSCs (Figure 2B). Ferroptosis is a controlled kind of cell death characterized by the iron-dependent buildup of lipid peroxides. GPX4 is an important regulator in this process. Thus, these findings shed light on the potential role of a GPX4-mediated ferroptosis mechanism in the cellular response to BP.

### BP-induced lipid peroxidation and reactive oxygen species (ROS) in ovarian cancer cells

**Figure 3:**
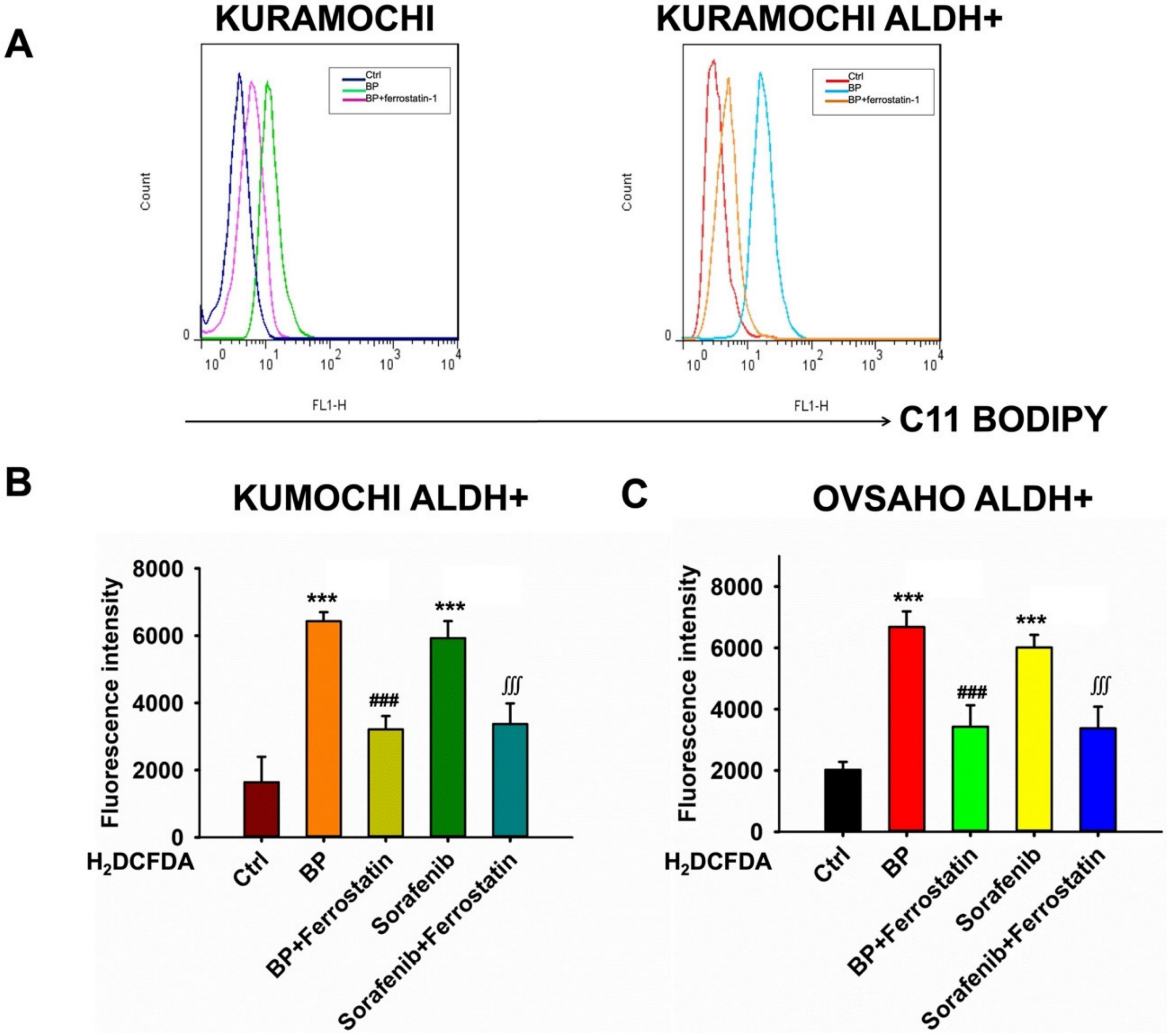
n-butylidenephthalide (BP) induced lipid peroxidation and reactive oxygen species (ROS) in ovarian cancer cells. (A) Lipid peroxidation (represented by C11-BODIPY) was increased after BP treatment and was reversed by adding ferrostatin. (B) The ROS levels of different treatments (BP 50 μg/ml, sorafenib 20 μM and ferrostatin 20 μM) on KURAMOCHI ALDH^+^ cells. H_2_DCFDA (2',7'-dichlorodihydrofluorescein diacetate, a ROS indicator) was measured after the treatment. ***p<0.001 vs control, ###p<0.001 vs BP, ∭p<0.001 vs sorafenib. (C) The ROS levels of different treatments on OVSAHO ALDH_+_ cells. After BP (50 μg/ml), sorafenib (20 μM) treatments, the H_2_DCFDA increased and could be reversed by adding ferrostatin (20 μM), which meant BP could induce ferroptosis. ***p<0.001 vs control, ###p<0.001 vs BP, ∭p<0.001 vs sorafenib.

Next, the effects of BP on lipid peroxidation and ROS production in ovarian cancer cells were examined, with a focus on KURAMOCHI ALDH+ and OVSAHO ALDH+ cells. BP causes lipid peroxidation in these ovarian cancer cells. Treatment with BP raised levels of C11-BODIPY, a lipid peroxidation marker, indicating membrane damage (Figure 3A). This impact of BP was prevented by adding ferrostatin, an inhibitor of ferroptosis, indicating that BP may induce this cell death pathway.

BP raises ROS levels in KURAMOCHI ALDH+ (Figure 3B) and OVSAHO ALDH+ cells (Figure 3C). H2DCFDA, a ROS indicator, was shown to be increased following treatment with BP or sorafenib (20 μM). Sorafenib was an anticancer drug acts by inhibiting kinases, which have been used in many cancer types including ovarian cancer. Ferrostatin reversed the effect, demonstrating ferroptosis' involvement. Overall, the findings imply that BP induces ferroptosis in ovarian cancer cells via increasing lipid peroxidation and ROS generation. This mechanism can be used for targeted therapy, particularly in ALDH+ ovarian tumors.

### Impact of siHMBOX1 on mRNA expression and cell proliferation in KURAMOCHI and KURAMOCHI ALDH+ cells

**Figure 4:**
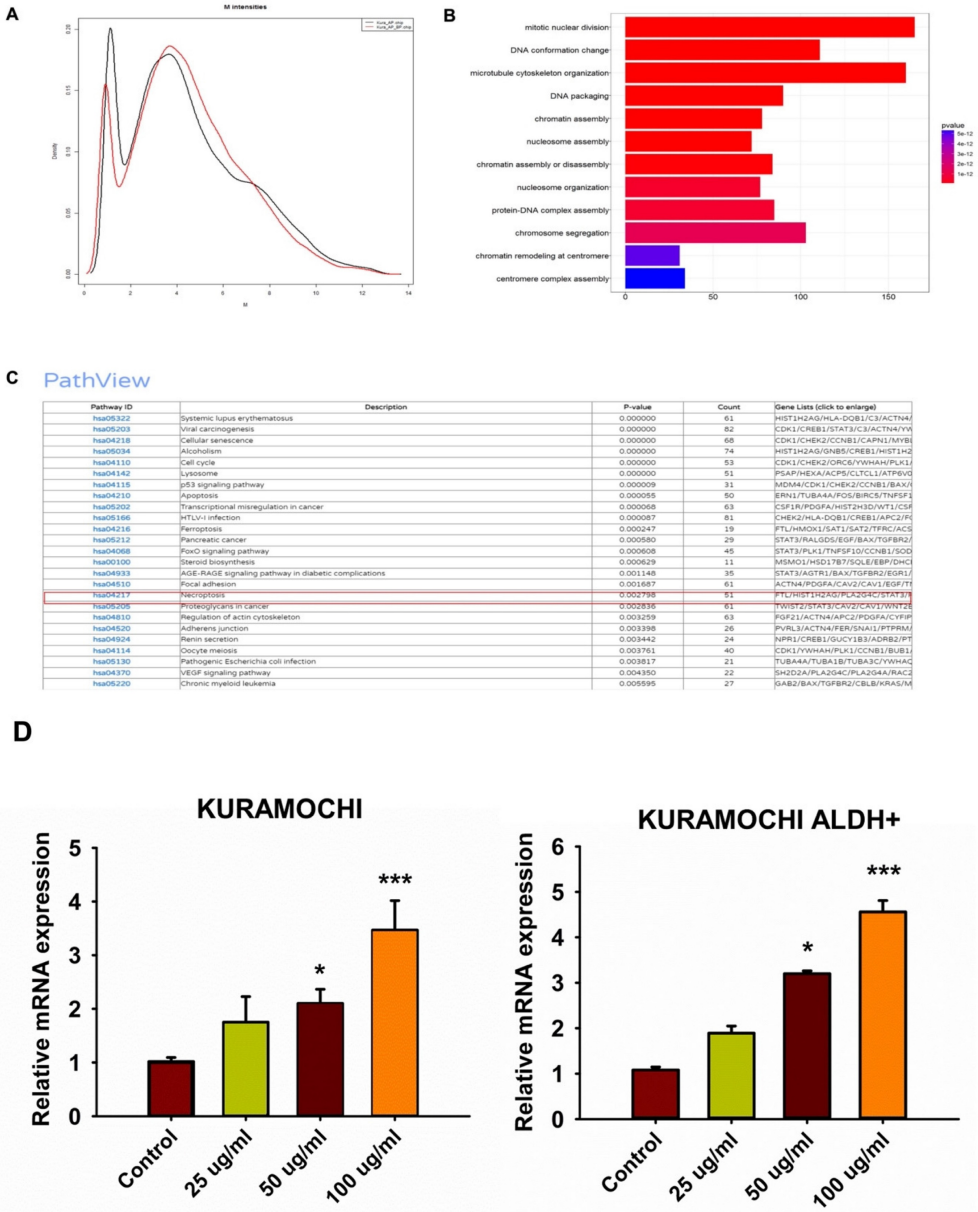
Up regulation of HMBOX-1 expression in BP treated KURAMOCHI cells. (A-C) gene expressions in BP treated KURAMOCHI cells were accessed with DNA microarray and GeneOntology analysis. Expression of ferroptosis-related genes including the *HMBOX-1* are a group of the major altered genes in the BP treated cells. (D) KURAMOCHI cells and the KURAMOCHI ALDH^+^ cells treated with BP (25, 50, 100 μg/ml). BP (100 μg/ml) significantly increased HMBOX-1 mRNA expression levels in the cells compared to the control group (*p<0.05, ***p<0.001 vs control).

**Figure 5:**
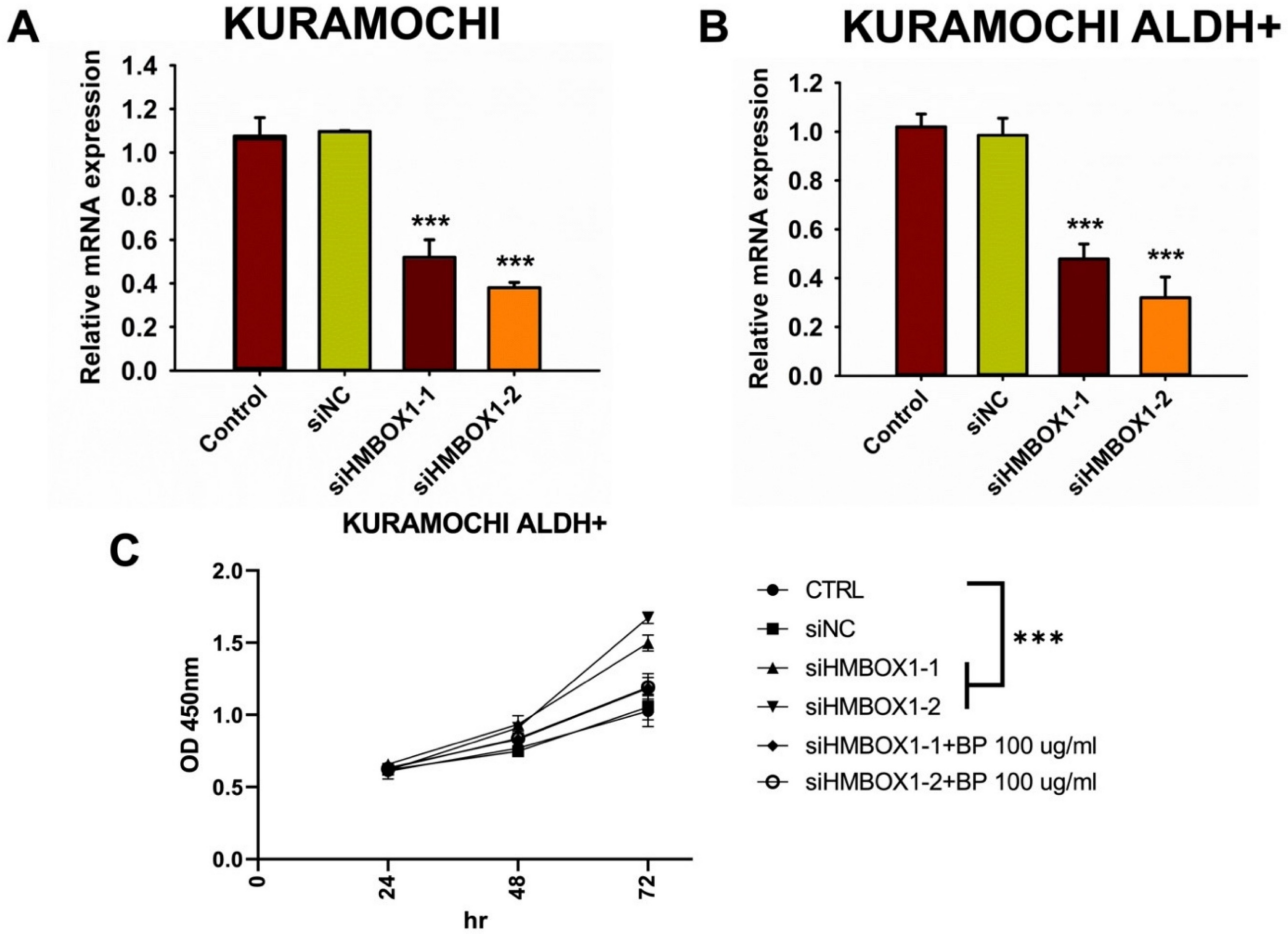
*HMBOX1* was involved in BP-inducing ferroptosis in ovarian cancer. (A) The analysis of mRNA expression levels of *HMBOX-1* in KURAMOCHI cells with or without siRNA of *HMBOX-1*. (B) The analysis of mRNA expression levels of *HMBOX-1* in KURAMOCHI ALDH^+^ cells with or without siRNA of *HMBOX-1*. (C) Cell proliferation curve of KURAMOCHI ALDH^+^ cells at various treatments, including siRNA of *HMBOX-1* and BP (100 μg/ml). ***P<0.001 vs control.

To investigate any other mechanisms that may be involved in BP-induced ferroptosis, gene expression in BP-treated cells was investigated using DNA microarrays. GeneOntology analysis revealed that BP had a significant impact on the expression of ferroptosis-related genes, including HMBOX-1 (heme oxygenase 1) (Figure 4A-C). HMBOX-1 regulates iron metabolism and is frequently increased during ferroptosis. The involvement of HMBOX-1 in BP function was studied further. BP (100 μg/ml) significantly increased HMBOX-1 mRNA expression levels in KURAMOCHI cells and KURAMOCHI ALDH+ cells compared to the control group (***p < 0.001) (Figure 4D). In cells treated with siHMBOX1-1 and siHMBOX1-2, HMBOX-1 mRNA expression levels were significantly lower compared to the control and siNC groups (***p < 0.001) (Figure 5A). Similar to KURAMOCHI cells, KURAMOCHI ALDH+ cells treated with siHMBOX1-1 and siHMBOX1-2 revealed a significant drop in relative mRNA expression of HMBOX-1 compared to the control and siNC groups (***p < 0.001; Figure 5B). At 72 hours, the siHMBOX1-1 and siHMBOX1-2 groups had considerably lower cell proliferation (OD at 450 nm) than the control (CTRL) and siNC groups (***p < 0.001). The addition of BP (100 µg/ml) did not significantly affect the decreased proliferation observed with siHMBOX1 treatments (Figure 5C). These findings suggest that HMBOX1 is involved in BP-induced cell death in ovarian cancer.

### Cell viability of cancer cells (OVSAHO) after BP and taxol treatments

**Figure 6:**
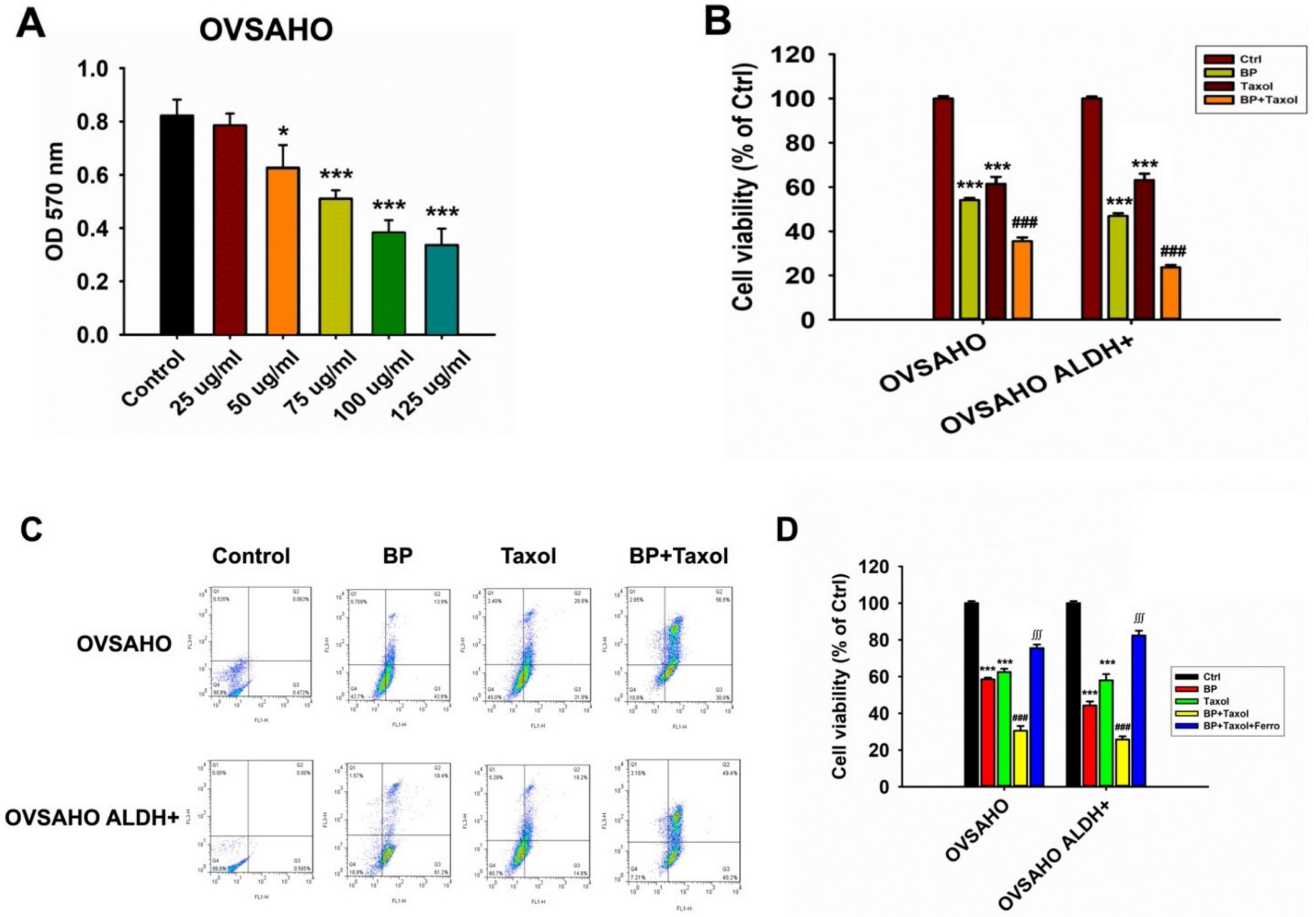
The cell viability of OVSAHO and OVSAHO ALDH+ cells treated with different concentrations of BP and Taxol. (A) Cell viability after treating different dosages of BP. BP reduced cell viability at a concentration from 25 to 125 µg/ml. *p<0.05, ***p<0.001 vs control. (B) Cell viability after treatment with BP (75 µg/ml), taxol (10 µM), and BP+taxol. ***p<0.001 vs control, ###p<0.001 vs BP and Taxol. (C) Flow cytometry of Annexin V revealed cell death percentage. (D) Cell viability after treatment with BP (75 µg/ml), taxol (10 µM), BP+taxol, and BP+taxol+ferrostatin. ***p<0.001 vs control, ###p<0.001 vs BP and Taxol, ∭p<0.001 vs BP+taxol.

The survival of OVSAHO cells after BP treatment was dose dependent. At BP concentrations above 50 µg/ml, cancer cell survival was dramatically reduced (Figure 6A). The cytotoxicity increased significantly when BP was combined with taxol (p<0.001, Figure 6B). Flow cytometry further revealed that OVSAHO cells' apoptosis percentage rose greater following BP + taxol treatment than with BP or taxol alone (Figure 6C). Adding ferrostatin dramatically reduced cell viability in BP plus taxol treated OVSAHO cells and OVSAHO ALDH+ cells (p<0.001, Figure 6D). In conclusion, ferroptosis played a role in the cell-killing process during BP + taxol treatment.

### BP inhibited xenograft tumor growth via ferroptosis

**Figure 7:**
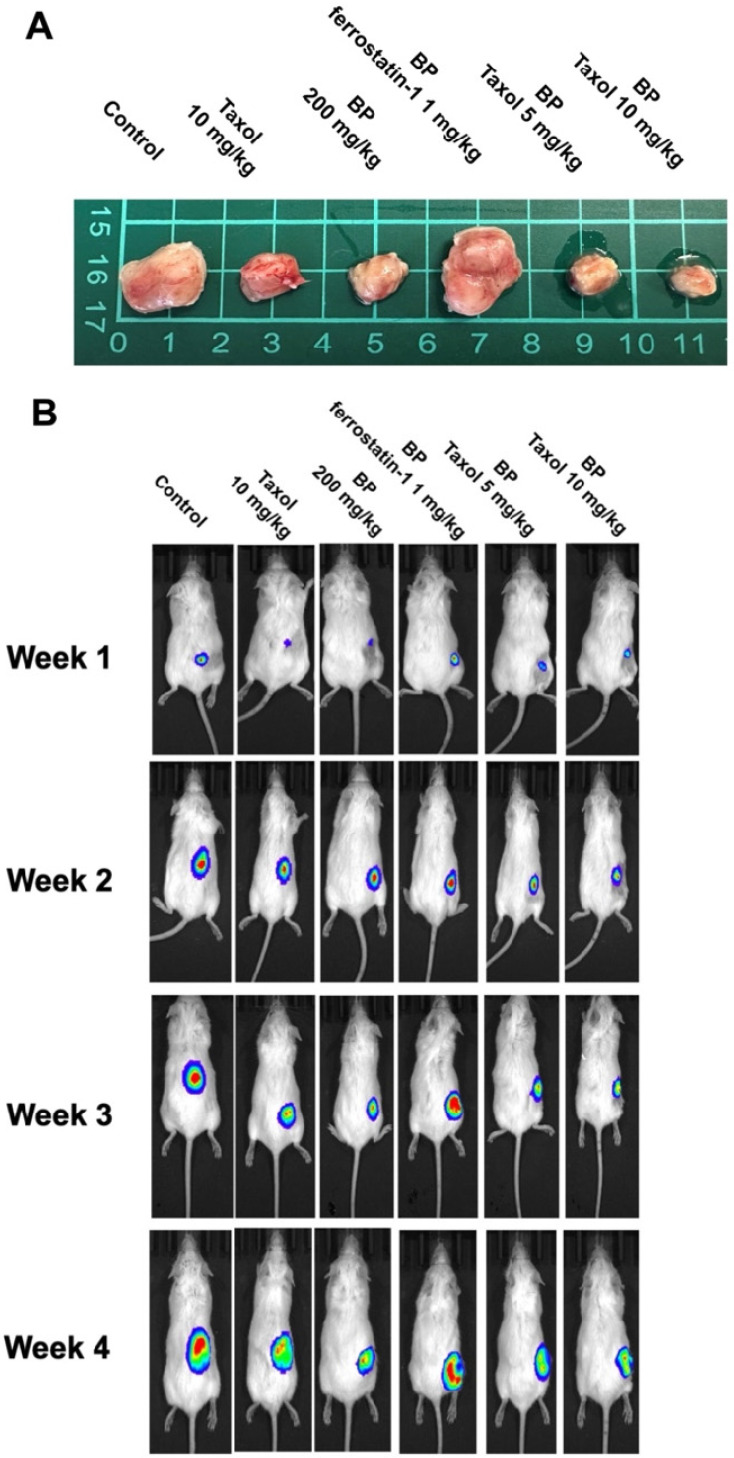

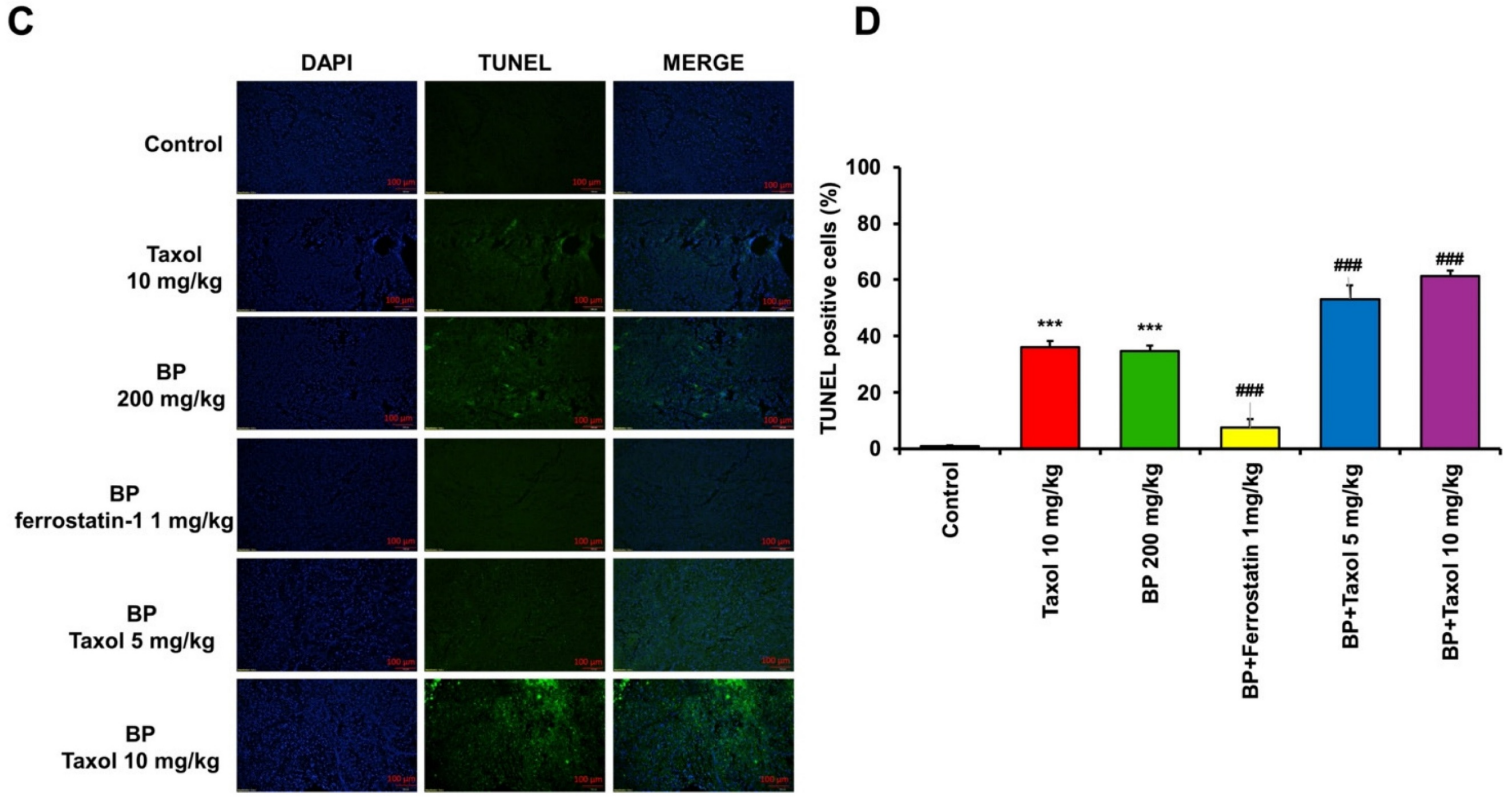
An *in vivo* investigation of n-butylidenephthalide (BP)-induced ferroptosis using NOD-SCID mice in a xenograft experiment. The experiment utilized KURAMOCHI ALDH^+^/luc cells. Both BP and taxol independently demonstrated the ability to inhibit tumor growth. (A) The upper panel displays the final tumor size of the xenograft, with a scale of 1 cm. (B) The lower panel depicts the tumor growth over the four weeks of the experiment. The growth-inhibitory impact of BP was nullified by the addition of ferrostatin-1 (a ferroptosis inhibitor). BP exhibited an adjuvant effect on Taxol, leading to a reduction in the Taxol dosage. The IVIS CT scan image represents each group, with a total of n = 3 in each group. (C) TUNEL assay of tumor tissue with or without BP and Taxol treatment. (D) Quantification of TUNEL positive cells in different treatment groups ***p<0.001 vs control, ###p<0.001 vs BP and Taxol.

To explore BP's anti-tumor impact *in vivo*, a xenograft cancer animal model was constructed using KURAMOCHI ALDH+/luc cells. Tumor size was assessed weekly for 5 weeks using an IVIS CT scan (Perkin Elmer, Waltham, MA, USA). The pictures were taken using the IVIS Spectrum CT and analyzed with the Living Image 4.4 software. D-luciferin (potassium salt, PerkinElmer Inc.) was delivered intraperitoneally to mice at a dose of 150 mg/kg prior to bioluminescence imaging. Following that, the animals were sedated with a mixture of oxygen and 2% isoflurane and placed in the imaging chamber. For 2D bioluminescence imaging (BLI), the device was designed to acquire images without employing an emission filter (open configuration). This was done to increase sensitivity and lower the detection threshold. Following the CT scan, a series of 2D bioluminescence surface radiance images were taken at different emission wavelengths. Both BP and taxol suppressed tumor growth separately. The administration of ferrostatin-1 reversed BP's growth inhibitory impact. BP and Taxol worked together to decrease tumor growth (Figures 7A and 7B). The TUNEL analysis of tumor tissue indicated significantly increased TUNEL+ cells after BP and taxol (10 mg/kg) treatment, which were decreased by ferrostatin-1 administration (Figure 7C). The results show that BP reduced type II ovarian cancer stem cell-derived tumor development by causing ferroptosis.

## Discussion

Ferroptosis is a kind of regulatory cell death (RCD) caused by the iron-dependent fatal accumulation of membrane-localized lipid peroxides, which is triggered by reactive oxygen species. Cells undergoing ferroptosis differ from other types of RCDs like as apoptosis, autophagy, cuproptosis, necroptosis, and pyroptosis. Ferroptotic cells accumulate ferrous ions and ROS, and they have dysmorphic tiny mitochondria with condensed membranes and reduced crista. The driving mechanism for cell death in ferroptosis has not yet been fully understood, however it is commonly believed that ferroptosis cell death occurs when the accumulation of oxidized phospholipids (PLs) and membrane permeability exceeds a particular threshold, leading to rupture 28-31. Cells, on the other hand, have many systems in place to prevent ferroptosis from occurring. The Glutathione peroxidase 4 (GPX4) antioxidant system is a key cellular defense mechanism against ferroptosis. GPX4 is an antioxidase that controls the availability of reduced glutathione (GSH) and effectively prevents lipid oxidation. Reduction of GPX4 expression in cells was demonstrated to cause cells to undergo ferroptosis 32, 33.

The importance of ferroptosis in tumor biology has received more attention in recent years. On the one hand, ferroptosis appears to be an intrinsic tumor-suppression mechanism. Cancer cells, on the other hand, might develop several methods to avoid ferroptosis and so encourage their proliferation. Furthermore, CSCs, which are often resistant to apoptosis and standard therapies, are very susceptible to ferroptosis 13, 14. These support ferroptosis-based therapy as a novel and realistic cancer treatment method.

Contemporary strategies for managing ovarian cancer are specific to individual patients based on tumor histology and staging. These strategies include debulking surgery, platinum-based and taxane chemotherapies (such as paclitaxel and docetaxel), angiogenesis inhibitors, poly ADP-ribose polymerase (PARP) inhibitors, and immunotherapies 34. Zhang et al. reported the correlation between ferroptosis and p53 in ovarian cancer 35. Mitochondrial alterations, aberrant ROS production, and potential ferroptosis contribute to increased chemosensitivity in human ovarian cancer 36. PARP inhibitors induce lipid peroxidation and ferroptosis in ovarian cancer cells through the downregulation of SLC7A11 in a p53-dependent manner, consequently inhibiting tumor cell growth 36. miR-424-5p exerts a negative regulatory effect on ferroptosis in ovarian cancer cells through the targeting of ACSL4. The downregulation of miR-424-5p resulted in an increase in ACSL4 expression, which serves as a positive mediator of ferroptosis 37. This present study was an attempt to elucidate the molecular mechanism of BP in inducing ferroptosis in ovarian cancer cells.

Natural products may be exploited to develop less harmful anti-tumor agents 38, 39. BP, a naturally occurring chemical obtained from A. sinensis, has being explored as a cancer therapy. It has been found to cause apoptotic cell death in many types of cancer cells via distinct mechanisms. BP inhibits AP-2α and telomerase activity in lung cancer cells, leading to apoptosis 40. BP regulates endoplasmic reticulum stress, resulting in apoptosis in prostate cancer cells, and stimulates Nur77 translocation from nucleus to cytoplasm, culminating in cytochrome c release and caspase-3-dependent apoptosis in hepatocellular carcinoma cells 21. In addition, BP increases REDD1 expression in gastric cancer cells, inhibiting the mTOR signal pathway 20. However, whether this phytochemical can promote ovarian cancer cell death by ferroptosis is unknown. This study tested if BP can reduce HGSOC via ferroptosis.

One of CSC's characteristics is its ability to self-renew and differentiate, which can aid in carcinogenesis, development, and advancement 41, 42. A small number of CSCs in ovarian cancer can influence tumor aggressiveness, treatment resistance, and disease recurrence. These are the characteristics of HGSOC, a type II ovarian cancer 5, 6. As a result, ovarian CSC targeting is critical for cancer treatment, and medicines that target both cancer cells and CSCs might yield significant therapeutic benefits 43. Ovarian CSCs have significant levels of ALDH 44, 45. In this study, we employed ALDH as a marker to differentiate CSCs from KURAMOCHI and OVSAHO ovarian cancer cells. These cancer cells and extracted CSCs were employed to investigate the anticancer function and mechanism of BP. According to our findings, BP reduced the viability of these ovarian cancer cells and CSCs by inducing ferroptosis. Furthermore, when BP and Taxol were administered in combination for treatment, they had a cooperative cell killing effect. Ferroptosis cell death was evidenced by inhibition of GPX4 expression, increase of ROS, and lipid peroxidation in BP-treated cells. The canonical ferroptosis involves these cellular mechanisms. Additionally, BP has been demonstrated to up regulate HMBOX-1 expression in cells, indicating a non-canonical ferroptosis. HMBOX-1 degrades haem and releases Fe2+, which promotes ferroptosis by increasing lipid peroxidation 46. In other words, BP increased the execution of ferroptosis while decreasing the cellular protective mechanism in cells. These findings suggest that BP could be a phytochemical for the ferroptosis-based therapy of ovarian cancer.

A xenograft tumor model was constructed using ovarian KURAMOCHI ALDH+/luc cells to evaluate BP's anticancer effect *in vivo*. These KURAMOCHI ALDH+/luc cells are stemness-enriched cancer cells, hence tumors produced from them are HGSOC. Both BP and Taxol inhibited tumor development. The addition of ferrostatin-1 counteracted BP's growth inhibitory effects. This finding suggested that BP inhibited tumor growth via a ferroptosis-dependent mechanism. In addition, similar with the *in vitro* studies, the combination of BP and Taxol demonstrated a cooperative anti-tumor activity. The TUNEL assay of tumor samples revealed a considerable increase in cell death following BP and taxol therapy.

According to Zhou et al., erastin has a remarkable ability to counteract the effects of overexpressed ATP binding cassette subfamily B member 1 (ABCB1), which leads to ferroptosis and makes ovarian cancer cells more susceptible to docetaxel chemotherapy 47. This suggests that erastin and docetaxel work together synergistically. Limited *in vitro* studies have demonstrated significant antitumor effects of ferroptosis in ovarian cancer; however, there is a lack of *in vivo* applicable ferroptosis inducers that could be developed as potential therapeutic agents 34. Taxol is a widely used chemotherapeutic drug for ovarian cancer 48. Taxol stabilizes microtubules, resulting in cell death and mitotic arrest. This medicine, however, is extremely toxic and can cause severe adverse effects such as hypertension, angioedema, and urticaria. As a result, acute toxicity prohibits patients from finishing the full treatment cycle 49. Several investigations on lowering the toxicity of chemotherapy medicines were conducted. Previous research investigated the use of proadifen, cucurbitacin B, and gold nanoparticles to make ovarian cancer cells more susceptible to chemotherapeutic drugs 50-52, allowing for lower doses of the damaging substances. Another issue with taxol-based ovarian chemotherapy is drug resistance, which is the leading cause of failure in the treatment of ovarian cancer with this chemotherapeutic 53. In this study, we looked at the combination of BP and taxol. This study found a cooperative anti-tumor impact of BP and taxol, implying that BP may help make cells more responsive to taxol. In line with this, a prior study found that GPX4 inhibition accelerated ferroptosis and increased ovarian cancer cell sensitivity to taxol 12.

## Conclusion

In summary, this study identifies BP as a promising phytochemical with multifaceted anti-tumor potential in HGSOC. Mechanistically, BP induces ferroptosis through both canonical (ROS accumulation and GPX4 inhibition) and non-canonical (HMBOX1-mediated) pathways, effectively targeting both bulk tumor cells and cancer stem cells. Notably, its combination with Taxol demonstrated a synergistic antitumor effect, enhancing chemotherapeutic efficacy while potentially overcoming key clinical challenges such as drug resistance and toxicity. These mechanistic insights, coupled with the observed therapeutic synergy, highlight BP as a strong candidate for integration into ferroptosis-based combination therapies. Collectively, the findings lay a strong foundation for further *in vivo* validation and clinical translation of BP as a novel adjunct in the treatment of HGSOC.

## Figures and Tables

**Figure 8:**
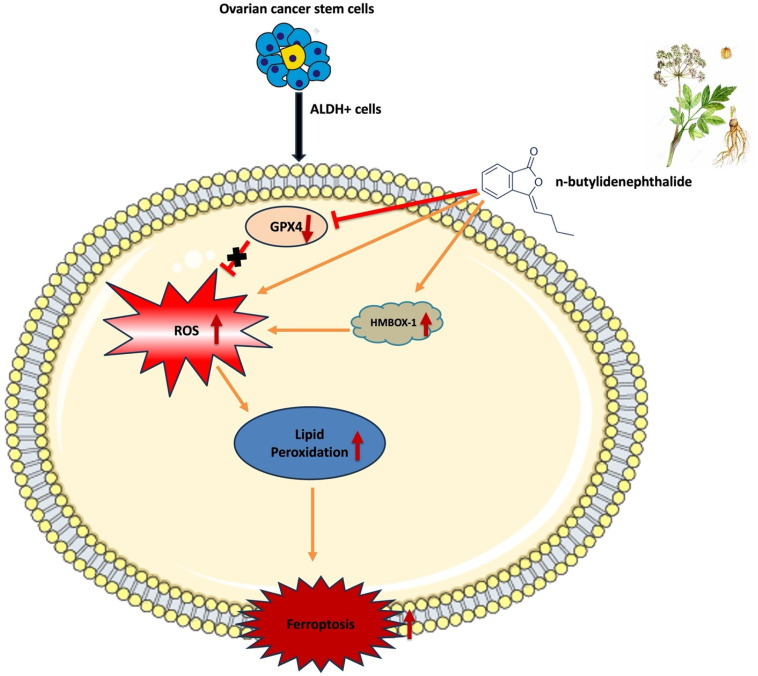
Schematic representation of mechanism of action of n-butylidenephthalide inducing ferroptosis in ovarian cancer cells

**Table 1:** Primer sequence used in the study

Gene Name	Forward (5'->3')	Reverse ((5'->3')	Base pair
*GPX4*	AGAGATCAAAGAGTTCGCCGC	TCTTCATCCACTTCCACAGCG	104
*GAPDH*	GGTCTCCTCTGACTTGAACA	GTGAGGGTCTCTCTCTTCCT	221

## Abbreviations

ALDH: aldehyde dehydrogenase
BP: n-butylidenephthalide
CSCs: cancer stem cells
HGSOC: high-grade serous ovarian cancer
FACS: fluorescence-activated cell sorting
FSP1: ferroptosis suppressor protein 1
GPX4: glutathione peroxidase 4
NEAA: non-essential amino acids
ROS: reactive oxygen species
TUNEL: terminal deoxynucleotidyl transferase (TdT) dUTP nick end labeling

## References

[1] Zhu B, Gu H, Mao Z, Beeraka NM, Zhao X, Anand MP (2024). Global burden of gynaecological cancers in 2022 and projections to 2050. J Glob Health.

[2] Teng YH, Liu FC, Huang SY, Kuo CF, Yu HP (2022). Epidemiology and Mortality of Ovarian Cancer in Taiwan: A Population-Based Study. J Clin Med.

[3] Pinsky PF, Yu K, Kramer BS, Black A, Buys SS, Partridge E (2016). Extended mortality results for ovarian cancer screening in the PLCO trial with median 15years follow-up. Gynecol Oncol.

[4] Bray F, Ferlay J, Soerjomataram I, Siegel RL, Torre LA, Jemal A (2018). Global cancer statistics 2018: GLOBOCAN estimates of incidence and mortality worldwide for 36 cancers in 185 countries. CA Cancer J Clin.

[5] Kurman RJ, Shih Ie M (2016). The Dualistic Model of Ovarian Carcinogenesis: Revisited, Revised, and Expanded. Am J Pathol.

[6] Koshiyama M, Matsumura N, Konishi I (2014). Recent concepts of ovarian carcinogenesis: type I and type II. Biomed Res Int.

[7] Kryczek I, Liu S, Roh M, Vatan L, Szeliga W, Wei S (2012). Expression of aldehyde dehydrogenase and CD133 defines ovarian cancer stem cells. Int J Cancer.

[8] Papaccio F, Paino F, Regad T, Papaccio G, Desiderio V, Tirino V (2017). Concise Review: Cancer Cells, Cancer Stem Cells, and Mesenchymal Stem Cells: Influence in Cancer Development. Stem Cells Transl Med.

[9] Silva IA, Bai S, McLean K, Yang K, Griffith K, Thomas D (2011). Aldehyde dehydrogenase in combination with CD133 defines angiogenic ovarian cancer stem cells that portend poor patient survival. Cancer Res.

[10] Monk BJ, Anastasia PJ (2016). Ovarian Cancer: Current Treatment and Patient Management. J Adv Pract Oncol.

[11] Giornelli GH (2016). Management of relapsed ovarian cancer: a review. Springerplus.

[12] Feng Q, Hao S, Fang P, Zhang P, Sheng X (2023). Role of GPX4 inhibition-mediated ferroptosis in the chemoresistance of ovarian cancer to Taxol *in vitro*. Mol Biol Rep.

[13] Zhang C, Liu X, Jin S, Chen Y, Guo R (2022). Ferroptosis in cancer therapy: a novel approach to reversing drug resistance. Mol Cancer.

[14] Zhou Q, Meng Y, Li D, Yao L, Le J, Liu Y (2024). Ferroptosis in cancer: From molecular mechanisms to therapeutic strategies. Signal Transduct Target Ther.

[15] Feng S, Tang D, Wang Y, Li X, Bao H, Tang C (2023). The mechanism of ferroptosis and its related diseases. Mol Biomed.

[16] Tang D, Chen X, Kang R, Kroemer G (2021). Ferroptosis: molecular mechanisms and health implications. Cell Res.

[17] Jiang Q, Wang K, Zhang X, Ouyang B, Liu H, Pang Z (2020). Platelet membrane-camouflaged magnetic nanoparticles for ferroptosis-enhanced cancer immunotherapy. Small.

[18] Tsai NM, Lin SZ, Lee CC, Chen SP, Su HC, Chang WL (2005). The antitumor effects of Angelica sinensis on malignant brain tumors *in vitro* and *in vivo*. Clin Cancer Res.

[19] Petrosyan E, Fares J, Fernandez LG, Yeeravalli R, Dmello C, Duffy JT (2023). Endoplasmic Reticulum Stress in the Brain Tumor Immune Microenvironment. Mol Cancer Res.

[20] Liao KF, Chiu TL, Huang SY, Hsieh TF, Chang SF, Ruan JW (2018). Anti-Cancer Effects of Radix Angelica Sinensis (Danggui) and N-Butylidenephthalide on Gastric Cancer: Implications for REDD1 Activation and mTOR Inhibition. Cell Physiol Biochem.

[21] Chen YL, Jian MH, Lin CC, Kang JC, Chen SP, Lin PC (2008). The induction of orphan nuclear receptor Nur77 expression by n-butylenephthalide as pharmaceuticals on hepatocellular carcinoma cell therapy. Mol Pharmacol.

[22] Chang YH, Lin YJ, Huang CY, Harnod T, Ding DC (2022). Shikonin impedes type 2 ovarian cancer progression via FasL/caspase-8 and mir-874-3p/XIAP axis and prohibits the properties of stemness. Am J Cancer Res.

[23] Leng Z, Yang Z, Li L, Zhong X, Zhou H, Li Y (2017). A reliable method for the sorting and identification of ALDH(high) cancer stem cells by flow cytometry. Exp Ther Med.

[24] Sebaugh JL (2011). Guidelines for accurate EC50/IC50 estimation. Pharm Stat.

[25] Chang YH, Liu HW, Chu TY, Wen YT, Tsai RK, Ding DC (2017). Cisplatin-Impaired Adipogenic Differentiation of Adipose Mesenchymal Stem Cells(1). Cell Transplant.

[26] Baskaran R, Chen Y-J, Chang C-F, Kuo H-N, Liang C-H, Abomughaid MM (2025). Potato protein hydrolysate (PPH902) exerts anti-lipogenesis and lipolysis-promoting effect by inhibiting adipogenesis in 3T3-L1 adipocytes. 3 Biotech.

[27] Hassannia B, Wiernicki B, Ingold I, Qu F, Van Herck S, Tyurina YY (2018). Nano-targeted induction of dual ferroptotic mechanisms eradicates high-risk neuroblastoma. The Journal of clinical investigation.

[28] Pope LE, Dixon SJ (2023). Regulation of ferroptosis by lipid metabolism. Trends Cell Biol.

[29] Zhou X, Hong Y, Zhang H, Li X (2020). Mesenchymal Stem Cell Senescence and Rejuvenation: Current Status and Challenges. Front Cell Dev Biol.

[30] Chen X, Yu C, Kang R, Tang D (2020). Iron Metabolism in Ferroptosis. Front Cell Dev Biol.

[31] Hassannia B, Vandenabeele P, Vanden Berghe T (2019). Targeting Ferroptosis to Iron Out Cancer. Cancer Cell.

[32] Conrad M, Friedmann Angeli JP (2015). Glutathione peroxidase 4 (Gpx4) and ferroptosis: what's so special about it?. Mol Cell Oncol.

[33] Krummel B, Plotz T, Jorns A, Lenzen S, Mehmeti I (2021). The central role of glutathione peroxidase 4 in the regulation of ferroptosis and its implications for pro-inflammatory cytokine-mediated beta-cell death. Biochim Biophys Acta Mol Basis Dis.

[34] Li L, Qiu C, Hou M, Wang X, Huang C, Zou J (2021). Ferroptosis in ovarian cancer: a novel therapeutic strategy. Frontiers in oncology.

[35] Zhang Y, Xia M, Zhou Z, Hu X, Wang J, Zhang M (2021). p53 promoted ferroptosis in ovarian cancer cells treated with human serum incubated-superparamagnetic iron oxides. International Journal of Nanomedicine.

[36] Gentric G, Kieffer Y, Mieulet V, Goundiam O, Bonneau C, Nemati F (2019). PML-regulated mitochondrial metabolism enhances chemosensitivity in human ovarian cancers. Cell metabolism.

[37] Lin-Lin M, Liang L, Dan Z, Shao-Wei W (2021). Tumor suppressor miR-424-5p abrogates ferroptosis in ovarian cancer through targeting ACSL4. Neoplasma.

[38] Asma ST, Acaroz U, Imre K, Morar A, Shah SRA, Hussain SZ (2022). Natural Products/Bioactive Compounds as a Source of Anticancer Drugs. Cancers (Basel).

[39] Ali Abdalla YO, Subramaniam B, Nyamathulla S, Shamsuddin N, Arshad NM, Mun KS (2022). Natural Products for Cancer Therapy: A Review of Their Mechanism of Actions and Toxicity in the Past Decade. J Trop Med.

[40] Wei CW, Lin CC, Yu YL, Lin CY, Lin PC, Wu MT (2009). n-Butylidenephthalide induced apoptosis in the A549 human lung adenocarcinoma cell line by coupled down-regulation of AP-2alpha and telomerase activity. Acta Pharmacol Sin.

[41] Liu M, Wu H, Xu C (2023). Targeting cancer stem cell pathways for lung cancer therapy. Curr Opin Oncol.

[42] Yang L, Shi P, Zhao G, Xu J, Peng W, Zhang J (2020). Targeting cancer stem cell pathways for cancer therapy. Signal Transduct Target Ther.

[43] Ma H, Tian T, Cui Z (2023). Targeting ovarian cancer stem cells: a new way out. Stem Cell Res Ther.

[44] Sharrow AC, Perkins B, Collector MI, Yu W, Simons BW, Jones RJ (2016). Characterization of aldehyde dehydrogenase 1 high ovarian cancer cells: Towards targeted stem cell therapy. Gynecol Oncol.

[45] Li Y, Chen T, Zhu J, Zhang H, Jiang H, Sun H (2018). High ALDH activity defines ovarian cancer stem-like cells with enhanced invasiveness and EMT progress which are responsible for tumor invasion. Biochem Biophys Res Commun.

[46] Sun Y, Chen P, Zhai B, Zhang M, Xiang Y, Fang J (2020). The emerging role of ferroptosis in inflammation. Biomed Pharmacother.

[47] Zhou H-H, Chen X, Cai L-Y, Nan X-W, Chen J-H, Chen X-X (2019). Erastin reverses ABCB1-mediated docetaxel resistance in ovarian cancer. Frontiers in oncology.

[48] Kampan NC, Madondo MT, McNally OM, Quinn M, Plebanski M (2015). Paclitaxel and Its Evolving Role in the Management of Ovarian Cancer. Biomed Res Int.

[49] Markman M, Kennedy A, Webster K, Kulp B, Peterson G, Belinson J (1999). Use of low-dose oral prednisone to prevent paclitaxel-induced arthralgias and myalgias. Gynecol Oncol.

[50] Jendzelovsky R, Jendzelovska Z, Hilovska L, Koval J, Mikes J, Fedorocko P (2016). Proadifen sensitizes resistant ovarian adenocarcinoma cells to cisplatin. Toxicol Lett.

[51] Chang YH, Wu KC, Ding DC (2021). The natural compound n-butylidenephthalide kills high-grade serous ovarian cancer stem cells by activating intrinsic apoptosis signaling pathways. J Cancer.

[52] Li S, Yang L, Wang J, Liang F, Chang B, Gu H (2016). Analysis of the chemotherapeutic effects of a propadiene compound on malignant ovarian cancer cells. Oncotarget.

[53] Wender PA, Galliher WC, Bhat NM, Pillow TH, Bieber MM, Teng NN (2012). Taxol-oligoarginine conjugates overcome drug resistance in-vitro in human ovarian carcinoma. Gynecol Oncol.

